# Incomplete Segregation of *MSH6* Frameshift Variants with Phenotype of Lynch Syndrome

**DOI:** 10.3390/ijms18050999

**Published:** 2017-05-06

**Authors:** Raffaella Liccardo, Marina De Rosa, Giovanni Battista Rossi, Nicola Carlomagno, Paola Izzo, Francesca Duraturo

**Affiliations:** 1 Department of Molecular Medicine and Medical Biotechnology, Federico II University Medical School, 80131 Naples, Italy; liccardo@dbbm.unina.it (R.L.); marina.derosa@unina.it (M.D.R.); paola.izzo@unina.it (P.I.); 2 Endoscopy Unit, Fondazione Pascale National Institute for Study and Care of Tumors, 80131 Naples, Italy; giorossi.alice@alice.it; 3 General Surgery Unit—Advanced Biomedical Science Department, Federico II University Medical School, 80131 Naples, Italy; nicola.anita@tiscali.it; 4 CEINGE-Biotecnologie Avanzate, 80145 Naples, Italy

**Keywords:** Lynch syndrome, segregation analysis, *MSH6* gene, hereditary colorectal cancer, oligogenic model

## Abstract

Lynch syndrome (LS), the most frequent form of hereditary colorectal cancer, involves mutations in mismatch repair genes. The aim of this study was to identify mutations in *MSH6* from 97 subjects negative for mutations in *MLH1* and *MSH2*. By direct sequencing, we identified 27 *MSH6* variants, of which, nine were novel. To verify the pathogenicity of these novel variants, we performed in silico and segregation analyses. Three novel variants were predicted by in silico analysis as damaging mutations and segregated with the disease phenotype; while a novel frameshift deletion variant that was predicted to yield a premature stop codon did not segregate with the LS phenotype in three of four cases in the family. Interestingly, another frame-shift variant identified in this study, already described in the literature, also did not segregate with the LS phenotype in one of two affected subjects in the family. In all affected subjects of both families, no mutation was detected in other *MMR* genes. Therefore, it is expected that within these families, other genetic factors contribute to the disease either alone or in combination with *MSH6* variants. We conclude that caution should be exercised in counseling for *MSH6*-associated LS family members.

## 1. Introduction

Lynch syndrome (LS) is the most common form of hereditary colorectal cancer (CRC) with an incidence of 3–5% among all cases of CRC, whereas its main genetic counterparts, familial adenomatous polyposis (FAP) and *MYH*-associated polyposis (MAP) syndromes, account for 1% of CRC diagnoses [1,2,3]. The main clinical manifestation of LS is the development of colon cancer on average around 45 years; this syndrome is also characterized by an increased risk of developing extra-colonic tumors, such as endometrial cancer, ovarian, stomach, urinary and biliary tract. Germline mutations in the DNA mismatch repair (*MMR*) genes are responsible for the LS phenotype [1]. Germline mutations in *MLH1* and *MSH2* account for about 40% and 39%, respectively, of all LS-associated mutations [4]. Approximately 10–20% of the mutations in *MMR* genes have been identified in *MSH6* and *PMS2* genes [5]; moreover, mutations in *MLH3* and *MSH3* genes [6,7] associated with LS phenotype were also reported. These mutations determine the loss of function of the MMR complex that, at the somatic level, is manifest as high levels of microsatellite instability (MSI), which occurs in >90% of all LS carcinomas [8]. The lost expression of the affected protein, at the somatic level, is also detectable by immunohistochemistry (IHC) analysis [9]. Identification of families affected by LS occurs by the Amsterdam Criteria (AC) and Bethesda Guidelines (BG) [10,11,12].

Identifying carriers of *MMR* mutations is critical for improving cancer surveillance and prevention. Until a few years ago, genetic testing was performed only for *MLH1* and *MSH2*; when these were negative, mutations in *MSH6* were investigated [4]. Recent guidelines recommend testing the tumors of all patients with CRC with either IHC or for MSI to identify potential cases of LS; moreover, all patients meeting AC or BL should be offered germline genetic testing for mutations in *MLH1*, *MSH2*, *MSH6* and *PMS2* [13].

*MSH6* is located on chromosome 2p16, near *MSH2* [14,15], and can cause an LS phenotype, although germline mutations have been attributed to families with incomplete penetrance, delayed cancer onset and low MSI [16,17,18]. Detecting mutations in *MMR* genes has been mainly carried out in Lynch patients with tumors with high MSI, which may explain the lower frequency of observed mutations in *MSH6*. Moreover, mutations in *MSH6* confer a relatively greater risk of endometrial cancer [19,20]. The aim of this study was to investigate the contribution of mutations in *MSH6* in 97 CRC patients belonging to 74 Lynch families, already negative for germline mutations in *MLH1* and *MSH2*. We report several molecular variants in our Lynch patients, of which nine are novel variants. Moreover, we describe two Lynch families in which *MSH6* variants do not fully segregate with the cancer phenotype and discuss the clinical implications of this finding.

## 2. Results

All *MSH6* exons were analyzed on DNA extracted from 97 CRC patients belonging to 74 families selected by the AC and BG. As shown in Table 1, 27 germline variants were identified in *MSH6*, nine of which were novel variants not previously reported in the NCBI SNP database, the Human Gene Mutation Database (available online: http://www.hgmd.cf.ac.uk/ac/index.php), the International Society for Gastrointestinal Hereditary Tumors (InSight; available online: http://www.insightgroup.org/) or the *MMR* variants database [21]. MSI analysis was performed for all patients with available paraffin-embedded tumor tissues; these results are listed in Table 1.

Missense mutations (30%) and intronic alterations (30%) were the most common aberrations. The other observed mutations were silent variants (26%), frame-shift variants (7%) and an in-frame insertion of three nucleotides (4%). Details on the prevalence of each variant are given in Table 1. The nine novel DNA variants were not detected in the 100 healthy controls from Southern Italy (Table 2). To verify the pathogenicity of the novel variants, we used a combination of computational and segregation analyses, as described in our previous studies [7,21]. The results are shown in Table 2.

**Figure 1:**
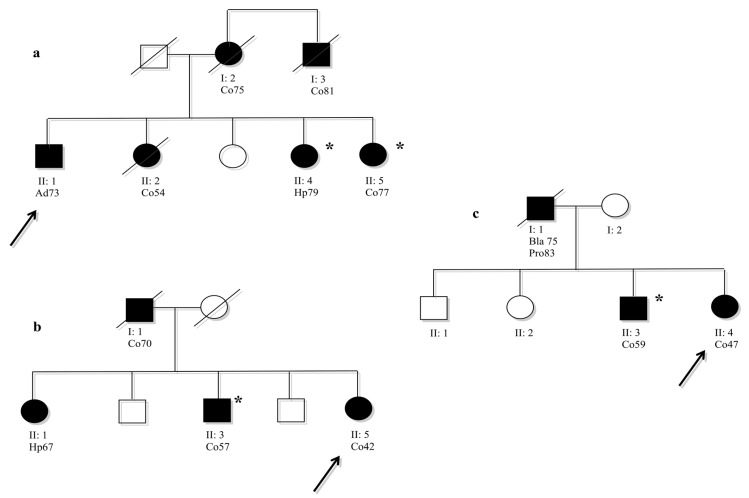
(**a**) Family 33: pedigree of index case (ID 409) carrier intronic variant c.457+33_+34insGTGT in exon 2 of *MSH6* gene; (**b**) Family 26: pedigree of the index case (ID 210) carrier missense variant c.1395 A>T in exon 4 of the *MSH6* gene; (**c**) Family 102: pedigree of the index case (ID 1454) carrier in the frame variant c.2049_2050insAGT in exon 5 of the *MSH6* gene. Symbols and abbreviations used are denoted as follows: arrow, index case; black symbol, cancers or colon adenomas associated with LS; Co, colon cancer; Ad, adenoma with moderate dysplasia; Hp, hyperplastic polyps; Bla, bladder cancer; Pro, prostate cancer. The number next to the diagnosis denotes age at onset. * Denotes family members are carriers of a specific variant.

**Figure 2:**
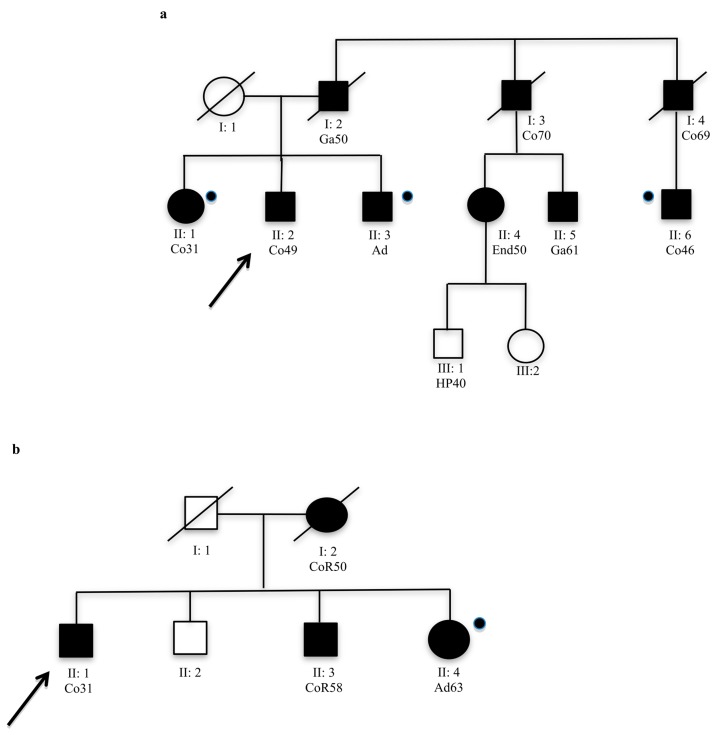
(**a**) Family 21 pedigree of the index case (ID 105) carrier of novel frameshift variant, c. 3296_97delTT in exon 5 of the *MSH6* gene; (**b**) Family pedigree of the index case (ID 103) carrier of known frameshift variant c. 3261dupC in exon 5 of the *MSH6* gene. Symbols and abbreviations used are denoted as follows: arrows, index case; black symbol, cancers or colon adenomas associated with LS; Co, colon cancer; CoR, colorectal cancer; Ga, gastric cancer; End, endometrial cancer; Ad, adenoma with severe dysplasia; HP, hyperplastic polyps. Numbers next to the diagnosis denote age at onset; 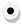 Analysed members that were not carried of mutation.

Computational analysis, in particular HSF, showed significant results for all novel variants identified in this study; while the segregation analysis (done only for 4/9 novel variants) did not always confirm the pathogenicity. The novel variants, c.457+33_+34insGTGT, c.2049_2050insAGT and c.2941A>G, segregated with the LS phenotype in the family (Figure 1). In contrast, the novel variant c.3296_97delTT in exon 5 of *MSH6*, identified in the index case No. 105 (Table 2), did not segregate with disease in the family (Figure 2a). However, this variant was a frameshift mutation that produced a premature stop codon, resulting in a truncated protein; therefore, it would be considered a pathogenic variant. This variant was not identified in the other affected family members that met the AC (Table 2); MSI testing performed on DNA extracted from tumor tissue of the index case showed an MSI-high (H) status, but MSI-H was also identified in tumor DNA from Subject II:6 who was not a carrier of this variant (Figure 2a). The other affected subjects from this family II:1 and II:3 were not carriers of this variant (Figure 2a).

**Figure 3:**
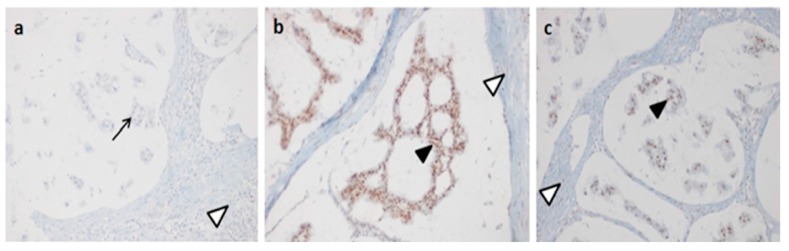
MSH6, MSH2 and MLH1 immunohistochemistry (IHC) results in the colon tumor section of the index Case 103 carrier of the c.3261dup variant in *MSH6* exon 5. (**a**) Absence of MSH6 protein in the tumor cells; (**b**) normal IHC for MSH2 protein in the tumor cells; (**c**) normal IHC for MLH1 protein in the tumor cells. Arrows point to IHC− tumor cells; filled arrow heads point to IHC+ tumor cells; open arrow heads point to blue nuclear staining of lymphocytes (positive internal control).

Interestingly, we identified another frameshift variant in exon 5 of *MSH6*, c.3261dup, that also produced an early stop codon and a truncated protein. This variant was already reported in the literature [22] and was classified as Class 5 in the Insight database. In our study, this variant was identified in the index case No. 103, a patient that developed CRC at age 61 (Figure 2b). MSI analysis showed an MSI-low (L); IHC showed the absence of MSH6 protein in tumor tissue (Figure 3), but segregation analysis showed that the variant was not present in the sister (II:4) of the index case, who also developed two colon adenomas, one of which with severe dysphasia (Figure 2b). We did not analyze other affected subjects in this family. For all affected individuals of these two families, carriers of *MSH6* frameshift mutations or not, the other *MMR* genes (*MLH3*, *MSH3* and *PMS2*) were sequenced, but no mutations were identified. Mutiplex Ligation Probe Ampification (MLPA) analysis of *MLH1*, *MSH2*, *MSH6* and *PMS2* in these individuals showed no deletions or duplications [23].

## 3. Discussion

Data from the literature showed that approximately 10% of families with LS were associated with mutations in *MSH6* [5]. In this study, we performed mutation analysis of *MSH6* in 97 patients with CRC belonging to 74 families selected by the AC and BG. For all patients that fulfilled the revised BG, an MSI analysis was performed using DNA extracted from tumor tissues. We identified 27 genetic variants, of which nine are novel. To identify the pathogenic effect of these novel variants, we used a multivariate analysis, as described in our previous studies [7,21]. In silico analysis showed a possible pathogenic effect for seven of the nine novel variants identified *MSH6* (Table 2). Segregation analysis, which is critical to understanding the contribution of a mutation to disease, does not always confirm computational data. Where it was possible, the segregation analysis was performed not only for novel variants, but for each variant identified in this study to verify the association with disease (Table 2). Interestingly, the novel variants, c.457+33_+34insGTGT, c.2049_2050insAGT and c.2941A>G, which were predicted to be pathogenic by in silico analysis, were also found to segregate with disease in their families (Figure 1). These three variants do not create a truncated protein, but probably alter the MSH6 protein, making it nonfunctional. However, the novel frame-shift variants (c.3296_97delTT in exon 5 of the *MSH6* gene) that yield a truncated protein were not found to segregate with a Lynch phenotype in either of their families. The novel frameshift mutation, c.3296_97delTT, was identified in a patient who developed colon cancer and belongs to a family that fulfilled the AC (Figure 2a). It was not possible to perform IHC on tumor tissue from this patient, but we were able to perform MSI analysis on tumor DNA, which showed a strong mutator phenotype. The same result was obtained from DNA extracted from colon tumor tissue from a cousin (II:6, Figure 2a) of our index case who was not a carrier of the *MSH6* mutation. Moreover, the sister and brother of our index case were not carriers of the frameshift variant; however, the sister developed a colon adenoma with a high degree of dysplasia at age 31, while the brother developed polyps at age 49. Therefore, we performed mutation detection analysis of other *MMR* genes (*MLH3*, *MSH3* and *PMS2*) for the index case and for all affected non-*MSH6* carriers belonging to the family. These patients were already known to be negative for mutations in *MLH1* and *MSH2*. No pathogenic mutations were identified. MLPA analysis of *MLH1*, *MSH2*, *MSH6* and *PMS2* in these individuals also showed no rare deletions or duplications [23]. Another frameshift mutation, c.3261dup, already described in the literature as pathogenic [22], was identified in our patient who developed colon cancer at age 61. This variant was not identified in the sister of the index case who developed two colon adenomas with severe dysplasia at age 63. We have not analyzed the other affected members of the family because they had died at diagnosis (Figure 2b). For this case, it was possible to perform IHC on paraffin-embedded tumor tissue from the index case. This analysis was negative for MSH6 protein expression (Figure 3), and MSI analysis showed MSI-L. Analysis of other *MMR* genes did not show pathogenic mutations.

Therefore, the *MSH6* frameshift variants in these two families could be not explain the onset of all cancers and/or adenomas, as other different genetic variants could be responsible for the LS phenotype observed in these families.

It is known that other Mendelian syndromes with autosomal-dominant inheritance patterns show an overlapping clinical presentation with LS. In this regard, in previous study by our group, one patient with a Lynch-like phenotype, negative for mutations in *MMR* genes, showed a germline mutation in *PTEN* [24], which was associated with disease in the family. Sometimes, also tumors with MSI-H status have shown germline genetic variants in genes, such as *POLE* and *POLD1* [25], *MYH* [26] and also in *PTEN* [27], beyond *MMR* variants.

However, these two variants identified in *MSH6* create a truncated protein, and thus, it is tempting to assume that there is an effect of these alterations. It remains unclear whether these two truncating mutations truly initiate tumorigenesis. Interestingly, in our study, the missense mutations and in-frame insertions were found to segregate with disease in carriers in the families and index-cases, and these mutations showed a typical LS phenotype with MSH-H.

It is known that the MSH2-MSH6 complex recognizes both base:base and single base insertion/deletion mismatches, whereas the MSH2-MSH3 complex insertion/deletion mismatches [28,29]. The major role of the MSH2-MSH6 complex also justifies the relative abundance compared to the MSH2-MSH3 complex in several species, such as yeast, human and mouse [30].

Almost 20 years ago, Edelmann et al. showed that mice homozygous for missense mutations in *MSH6* were unable to repair any type of defect, because the mutated protein competes with the wild-type protein in the formation of the MSH2-MSH6 complex [31]. Meanwhile, subsequent studies have shown that truncated proteins caused by frameshift mutations yielded a weak mutator phenotype [32]. This suggested that tumors with missense mutations in *MSH6* show a more severe phenotype because the presence of mutant MSH6 protein in tumor cells interferes with MSH2-MSH3-mediated repair. In contrast, a lack of MSH6 protein does not interfere with the function of the MSH2-MSH3 heterodimer. In cases where MSH6 is not expressed (null mutations), a severe phenotype, if present, is likely to be due to a secondary mutation in an *MMR* gene, such as *MSH3*. Taking previous data into consideration, the *MSH6* variants could be responsible for part of the LS phenotype, and additional genetic factors could lead to the disease either by themselves or in combination with the *MSH6* mutations; thus operating in a di- or even multi-genic model [7,33,34]. The theory of oligogenic disease would also explain the generally variable onset and severity, as well as the reduced penetrance between and within *MSH6* families. Interestingly, the index Case 210 of Family 26 was a carrier of two *MSH6* variants: the missense variant, c.2941A>G, also identified in her brother (Figure 1b), and the silent variant, c.1395A>T, that was not associated with disease in the family. Perhaps for this reason, this subject showed a severe phenotype compared to the other affected members in the family.

In summary, we present several variants in *MSH6*. The mutations that do not delete the protein seem to be associated with a typical LS phenotype, while incomplete segregation of two *MSH6* frameshift variants in two independent families may suggest that additional genetic factors are involved in the etiology of the disease, possibly acting as an oligogenic model. High-throughput sequencing technologies may help to uncover the genetic basis of LS in the families described in this report [35].

Genetic counseling and specialized monitoring of families with inherited forms of LS is crucial. Identification of a causal gene in the family will have implications for screening and endoscopic surveillance [1,2,36,37]. Therefore, family members who do not carry these *MSH6* frameshift variants cannot be considered healthy and should still participate in specialized surveillance programs. Finally, this study remarks that segregation analysis remains a very important tool in clinical genetics.

## 4. Patients and Methods

### 4.1. Patients

The patients were recruited from several hospitals in Campania (Southern Italy). Seventy-four subjects with Lynch syndrome diagnosed by the AC or BG and negative for mutations in *MLH1* and *MSH2* were selected. As negative controls, 100 samples from healthy Caucasian patients were collected from the Clinical Department of Laboratory Medicine of the hospital affiliated with Federico II University (Naples, Italy).

Samples from all subjects were collected after being granted authorization from the local ethics committee “Comitato etico per le attività Biomediche Carlo Romano” of the University of Naples, Federico II (Protocol No. 120/10; Date: 15 September 2010). Once the authorization was obtained, the study received ethical approval, and participants’ informed and written consent was obtained. The experiments were performed on DNA extracted from peripheral blood lymphocytes and from paraffin-embedded tumor tissues. For the healthy samples, DNA was extracted only from peripheral blood lymphocytes.

### 4.2. Isolation of Genomic DNA

Total genomic DNA was extracted from 4 mL peripheral blood lymphocytes using a BACC2 Nucleon kit (Amersham Pharmacia Biotech, Amersham, UK). For each paraffin block, five 20-μm sections were cut and collected in a 1.5-mL micro-tube. DNA was extracted after deparaffinization according to the protocol described by Duraturo et al., 2015, and using a BACC2 Nucleon kit (Amersham Pharmacia Biotech) [38].

### 4.3. DNA Amplification and Microsatellite Analysis

MSI was tested on paired samples of lymphocyte DNA and DNA from paraffin-embedded tumor sections. MSI was evaluated with a fluorescent multiplex system comprising five mononucleotide repeats (BAT-25, BAT-26, NR-21, NR-24 and NR-27), three dinucleotide repeats (D2S123, D5S346 and D17S250) and two tetranucleotide repeats using the CC-MSI kit (AB ANALITICA, Padova, Italy) and subsequent capillary electrophoresis analysis using an ABI 3130 Prism (Applied Biosystems, Thermo Fisher Scientific, Waltham, MA, USA). Tumors were classified as “highly unstable” (MSI-H), if at least 30% of the markers showed instabilities, and as “low-level instability” (MSI-L), if at least 10% of the markers showed instabilities; if no allele differences between DNA extracted from normal and tumorous tissues were observed, tumors were classified as microsatellite stable (MSS) [39,40,41].

### 4.4. Mutation Analysis

Amplification, denaturing high-performance liquid chromatography (dHPLC) and sequencing were all performed using standard protocols. All *MSH6* exons were amplified, including intron-exon boundaries, from DNA extracted from blood lymphocytes of 97 patients, using customized primer sets. Prior to dHPLC analysis, the polymerase chain reaction (PCR) products were separated on a 1–2% agarose gel to check for unspecific amplicons. A Transgenomic Wave DNA Fragment Analysis system (3500 HT; Transgenomic, Inc., Omaha, NE, USA) was used to perform dHPLC analysis. For all samples exhibiting abnormal dHPLC profiles, genomic DNA was re-amplified and sequenced in the forward and reverse directions using an ABI 3100 Genetic Analyser (Applied Biosystems).

### 4.5. In Silico Analysis

Structural analysis of point variants is important to understand the functional activity of the mutated protein. We used three complementary algorithms for functional impact prediction of the novel variants: Sorting Intolerant From Tolerant (SIFT) (available online: http://blocks.fhcrc.org/sift/SIFT.html) [42], Polymorphism Phenotyping (PolyPhen) (available online: http://genetics.bwh.harvard.edu/pph/) [43] and Human Splicing Finder (HSF) (available online: http://www.umd.be/HSF/) [44], as described in our previous studies [7,38].

### 4.6. Immunohistochemistry

IHC was performed on a Benchmark XT automatized immunostainer (Ventana Medical Biosystems, Tucson, AZ, USA). The antibodies used were anti-MSH6, mouse monoclonal clone 44, anti-MSH2, mouse monoclonal clone G219-1129, and anti-MLH1, mouse monoclonal clone M1 (Ventana Medical Biosystems). The detection system used was an iVIEW DAB Detection Kit (Ventana Medical Biosystems), which is based on the streptavidin-biotin-conjugated system. Nuclear staining was observed with an optical microscope positivity represented by the presence of brown staining. This positivity was compared with blue nuclear epitopes, in which the specific antigen was not present. The internal positive control was represented by lymphocytes, stroma and functional mucosal crypts, while the negative control was obtained by slides without primary antibody. Nuclear immunoreactivity scores were assigned using range from 0–100%.

## 5. Conclusions

In this study, we have identified nine novel variants in *MSH6* gene which enlarge the mutation spectrum of this gene. Furthermore, the incomplete segregation of two *MSH6* variants in two independent families may suggest that additional genetic factors are involved in the etiology of the disease, possibly acting as an oligogenic model. Finally, our data showed that segregation analysis remains important in clinical genetics.

## Figures and Tables

**Table 1:** Variants identified in the *MSH6* gene in our study.

Exon	Nucleotide Change	Amino Acid Change	Frequency in Hereditary CRC	Reference (Reportage)	Other Studies		
					Segregation Analysis	MSI	IHC
1	c.116 G>A	p.Gly39Glu	42 families	dbSNP-rs1042821	ND	ND	ND
1	c.186 A>C	p. = (Arg)	40 families	Nicolaides et al. 1996 (29 times)	ND	ND	ND
1	c.260 +22 C>G		29 families	Kolodner et al. 1999 (8 times)	ND	ND	ND
2	c.261 −46 A>G		1 family	this study	ND	ND	ND
2	c.276 A>G	p. = (Pro)	20 families	dbSNP-rs1800932	ND	ND	ND
2	c.431 G>T	p.Ser144Ile	1 family	Wu et al. 1999 (26 times)	ND	MSI-H	ND
2	c.457 +33_+34insGTGT		1 family	Identified in this study	(+)	MSI-L	ND
2	c.457 +50 T>A		1 family	Identified in this study	ND	MSI-H	ND
2	c.457 +52 T>A		3 families	Plaschke et al. 2000 (25 times)	ND	ND; ND; MSI-H	ND
3	c.540 T>C	p. = (Asp)	11 families	dbSNP-rs1800935	ND	ND	ND
4	c.642 C>T	p. = (Tyr)	6 families	Wijnen et al. 1999 (26 times)	ND	ND	ND
4	c.663 A>C	p.Glu221Asp	1 family	Devlin et al. 2008 (7 times)	ND	ND	ND
4	c.990 A>T	p. = (Ser)	1 family	Identified in this study	ND	MSI-H	ND
4	c.1164 C>T	p. = (His)	1 family	Kolodner et al. 1999 (4 times)	(−)	MSI-H	ND
4	c.1395 A>T	p. = (Ala)	1 family	Identified in this study	(−)	MSI-H	ND
4	c.2049_2050insAGT	p.Ala683_Leu684insSer	1 family	Identified in this study	(+)	MSI-H	MSH6-; MSH2+; MLH1+
4	c.2398 G>C	p.Val800Leu	1 family	Kolodner et al. 1999 (3 times)	(+)	MSI-H	ND
4	c.2633 T>C	p.Val878Ala	2 families	dbSNP-rs2020912	ND	MSI-H, ND	ND
4	c.2941 A>G	p.Ile981Val	1 family	Identified in this study	(+)	MSI-H	ND
5	c.3226 C>T	p.Arg1076Cys	1 family	Plaschke et al. 2000 (8 times)	ND	MSI-H	ND
5	c.3261dup	p.Phe1088Argfs*3	1 family	Bonk et al. (2 times)	(−)	MSI-L	MSH6-; MSH2+; MLH1+
5	c.3296_3297delTT	p.Ile1099delinsAsnfs*8	1 family	Identified in this study	(−)	MSI-H	ND
5	c.3438 +14 A>T		15 families	dbSNP-rs2020911	ND	ND	ND
7	c.3639 T>A	p.Asp1214Glu	1 family	Identified in this study	ND	ND	ND
7	c.3646 +31_+34del		16 families	dbSNP-rs1805181	ND	ND	ND
8_9	c.3802−42insT		4 families	Plaschke et al. 2000 (2 times)	ND	ND; ND; ND; ND	ND
8_9	c.3801 +54C>G		8 families	Kolodner et al. 1999 (10 times)	ND	ND	ND

NCBI accession number: NM000179; CRC: colorectal cancer; ND: not done; MSI-L/H: low/high microsatellite instability; IHC: immunohistochemistry.

**Table 2:** Novel MSH6 variants identified in this study.

Family	ID	Mutation	Protein Effect	In Silico Analysis			Frequency in Healthy Controls	Phenotype MSI	Segregation Analysis in Affected Subjects
				PolyPhen (Score)	SIFT (Score)	HSF			
31	808	Ex2 c.261 -46 A>G		ND	ND	+3’ss; +BP	0/100	AM−; ND	ND
33	409	Ex2 c.457+33_+34insGTGT		ND	ND	+3’ss × 2; +ESE	0/100	AM−; MSI-L	3/3
10	9529	Ex2 c.457 +50 T>A		ND	ND	+3’ss	0/100	AM+; MSI-H	ND
34	410	Ex4 c.990 A>T	p. = (Ser)	ND	ND	−SRp55; −EIE	0/100	AM+; MSI-H	ND
26	210	Ex4 c.1395 A>T	p. = (Ala)	ND	ND	−SRp55; +ESS × 2; +ESR	2/100	AM+; MSI-H	1/2
102	1454	Ex4 c.2049_2050insAGT	p.Ala683_Leu684insSer	ND	ND	+3’ss × 2; +BP	0/100	AM−; MSI-H	2/2
26	210	Ex4 c.2941 A>G	p.Ile981Val	Benign (0.181)	Tolerated (1)	+3’ss; +ESE × 2; −EIE × 2; +ESS; +9G8; +ESR	0/100	AM+; MSI-H	2/2
21	105	Ex5 c.3296_97delTT	p.Ile1099delinsAsnfs*8	ND	ND	ND	0/100	AM+; MSI-H	1/4
18	013	Ex7 c.3639 T>A	p.Asp1214Glu	Probably damaging (1)	Damaging (0)	+3’ss × 2; +ESE × 5; +EIE; +Tra2β; −IIE × 3; +ESR	0/100	AM+; ND	ND

ID: identification number patient; AM: Amsterdam Criteria; MSI-L/H: low/high microsatellite instability; ND: not done; motifs identified (+) or broken (−) by HSF (Human Splicing Finder): 3’ss, acceptor cryptic splice site; BP: branch point; ESE: exonic splicing enhancer; EIE: exon identity element; ESS: exonic splicing silencer; ESR: exonic splicing regulatory; IIE: intron identity element; SRp55, 9G8 and Tra2β: splicing enhancer proteins; SIFT: Sorting Intolerant From Tolerant. When multiple adjacent sites were predicted, the number of sites is indicated: ×2 means that two adjacent sites were modified by the mutation.
